# A Three-Year Experience With Overseas Kidney Transplantation in a Tertiary Transplant Center in Saudi Arabia

**DOI:** 10.7759/cureus.23988

**Published:** 2022-04-09

**Authors:** Mohammed Tawhari, Mansoor Radwi

**Affiliations:** 1 Nephrology, King Abdulaziz Medical City, Riyadh, SAU; 2 Nephrology, King Abdullah International Medical Research Center, Riyadh, SAU; 3 Nephrology, King Saud Bin Abdulaziz University for Health Sciences, College of Medicine, Riyadh, SAU; 4 Department of Hematology, University of Jeddah, College of Medicine, Jeddah, SAU

**Keywords:** immunosuppressive drugs, renal donor, graft rejection, kidney transplant recipient, end stage kidney disease (eskd)

## Abstract

Background

Overseas kidney transplantation is known to be associated with adverse outcomes. In this study, we aim to present a detailed analysis of our three years of experience with overseas kidney transplantation at one of the largest kidney transplant referral sites in the Kingdom of Saudi Arabia.

Materials and methods

A retrospective cohort study included patients who underwent kidney transplantation overseas and were subsequently followed up at King Abdulaziz Medical City from January 2016 to July 31, 2019. In addition, we compared the outcomes of the patients who underwent kidney transplantation overseas with a cohort of patients who were transplanted locally within the same period. Patients in both cohorts had to have at least one year of follow-up post-transplantation.

Results

We included a total of 51 patients who underwent kidney transplantation overseas. The mean age of the cohort was 44.7 years, and 69% were male. Almost 60% of the cohort had one or no comorbidity prior to transplant, with hypertension (84%) and diabetes mellitus (37%) being the leading comorbidities. The cause of end-stage kidney disease was unknown in 55% of our patients. In those who had an identifiable cause, lupus nephropathy and diabetes were the most common causes of kidney failure. In comparison with the locally transplanted cohort, no difference was detected between these groups in their baseline characteristics, type or number of comorbidities, medical or surgical complications postoperatively, and one-year mortality. However, we found that the graft rejection rate was significantly higher in patients transplanted overseas (OR=5.4, p<0.001). In addition, the proportion of patients who received anti-thymocyte globulin (ATG) induction was also less in the group with overseas kidney transplantation (58% vs. 22%, p<0.001).

Conclusion

Overseas transplantation is associated with an increased risk for graft rejection. Our study suggests that overseas kidney transplantation is possibly driven by a lack of donors, especially cadaveric. Counseling patients about risks associated with overseas kidney transplantation and encouraging the public to register for organ donation after death may help curb out this practice.

## Introduction

Patients with end-stage kidney disease (ESKD) suffer from a profound effect on their physical, psychological, and financial aspects due to morbidity caused by kidney failure and the requirement of chronic dialysis [1]. For most patients, kidney transplantation is the optimal treatment. It is well-known that most patients who undergo kidney transplantation experience improvement in quality of life and improved survival when compared to patients who are waiting for kidney transplantation [2]. One of the major obstacles to kidney transplantation is the availability of a kidney donor, whether living or deceased [3]. The scarcity of such donations has led to an increasing number of patients waiting for a kidney transplant. In 2016, a hundred thousand patients were found to be on the waiting list for a kidney transplant in the United States of America. In the same year, only nineteen thousand kidney transplants were performed [4]. The mean number of kidney transplants performed in Saudi Arabia between 2008 and 2016 was 129 transplants [5]. The number of transplants per year remained constant, yet the number of patients on dialysis increased during the same period. In 2016, there were 16,315 patients registered as dialysis-dependent, but only 2,708 patients (16.6%) were on the waitlist for a deceased donor kidney transplant. Also, the waiting list remained constant, with an average waiting time of 5.2 years to receive a deceased donor kidney transplantation [5]. Unfortunately, not all patients with ESKD will undergo transplantation in their lifetime.

The considerable waiting time has resulted in patients seeking transplantation in countries where barriers to transplant are less, and finding non-related living donors is feasible. Concerns in the medical community have risen due to this trend [6,7]. Many reports have concluded that transplant tourism or commercial transplant has not delivered the positive outcomes expected after kidney transplantation. Several reports have noticed an increase in infection rates and other comorbidities in patients who had kidney transplant overseas compared to locally performed transplantation [8-10]. Alghamdi and his colleagues published a local study in 2010 that compared outcomes between overseas and locally transplanted patients between 2003 and 2008 in Saudi Arabia [11]. The study found a higher rate of acute rejection in the first year, a higher mean creatinine at six months and one year, and a higher rate of cytomegalovirus infection and hepatitis C seroconversion among the recipients of overseas transplants.

In this paper, we present our experience with transplant tourism and provide information on trends, outcomes, and challenges associated with this practice. In addition, we hypothesize that the recipients of overseas transplant would have more comorbidities, would show an inferior graft outcome, and have a higher incidence of post-transplant complications. To prove our hypothesis, we compared the overseas kidney transplant cohort with another cohort of patients that underwent kidney transplantation locally within the same period.

## Materials and methods

The study was conducted at King Abdulaziz Medical City (KAMC) in Riyadh, Saudi Arabia. We included patients who underwent kidney transplantation overseas from January 2016 to July 31, 2019, with at least one year of follow-up post-transplantation. The recipients in this cohort underwent the transplant overseas at their own risk and without the consent/endorsement of their treating nephrologists. Consistent with the declaration of Istanbul on organ trafficking and transplant tourism, our center does not support transplant tourism, and such practice is considered exploitation of poor donors by rich individuals and is prohibited [12]. However, our center does not deny access to care for recipients of transplant tourism. 

We obtained the IRB approval from King Abdullah International Medical Research Center (RC20/386/R) before the initiation of the study. We collected baseline characteristics (e.g., age, gender, comorbidities prior to transplantation), transplant-related variables (e.g., immunosuppressive agents used, delayed graft function, kidney function at the time of discharge after transplantation), and post-transplant complications (e.g., post-transplant diabetes). 

We compared the baseline characteristics and outcomes of the cohort that underwent kidney transplantation overseas to a cohort of patients that were transplanted locally within the same period and who also had at least one year of follow-up post-transplantation. The details of the locally transplanted cohort were published separately earlier this year [13]. Due to better human leukocyte antigen (HLA) matching between the first-degree relatives, we anticipated that the graft-related outcomes were likely to be superior in our locally transplanted group, given that most of them received living-related kidney [13]. To diminish the HLA influence, we performed a subgroup analysis where we restricted the locally transplanted cohort to unrelated donors, whether deceased or living, and repeated the comparison to confirm if differences in graft-related outcomes remained superior in the locally transplanted group. 

Statistical Package for the Social Sciences (SPSS version 20; IBM Inc., Armonk, USA) software was used for data analysis. Categorical variables were presented as frequencies and percentages, whereas continuous variables were presented as mean ± standard deviation. We used the chi-squared test or Fisher's exact test to compare dichotomous data. Independent sample t-test analysis was applied to determine whether there was a statistical difference between the two groups' means. Odds ratio (OR) was used to estimate the magnitude of the association between post-transplant clinical outcomes and the location of transplantation.

## Results

We identified 51 patients who underwent kidney transplantation overseas and met our inclusion criteria. Their median age was 41 years (range 23-87), and their median pre-transplant BMI was 26 kg/m^2^ (range 16-35). The baseline characteristics of our cohort are presented in Table 1. 

**Table 1:** Baseline characteristics ^†^Other - chronic obstruction, reflux nephropathy, malignancies, and atrophied kidneys.

Variable	N	%
Gender (male)	35	68.6%
Comorbidities		
Diabetes mellitus	19	37.3%
Hypertension	43	84.3%
Dyslipidemia	12	23.5%
Stroke	0	0%
Cancer	1	2.0%
Amputation	1	2.0%
Coronary artery disease	4	7.8%
Congestive heart failure with reduced ejection fraction <55%	9	17.6%
Fracture prior to transplant	1	2.0%
Number of comorbidities		
No comorbidity	5	10.0%
One	25	50.0%
Two	9	18.0%
Three or more	11	22.0%
Renal replacement modality		
Hemodialysis via central venous catheter	26	51%
Hemodialysis via arteriovenous fistula/graft	10	19.6%
Preemptive	11	21.6%
Peritoneal dialysis	4	7.8%
Causes of end-stage kidney disease		
Idiopathic/unknown	28	54.9%
Systemic lupus erythematosus/glomerulonephritis	6	11.8%
Diabetes mellitus	6	11.8%
Hypertension/atrophied kidney	4	7.8%
Hereditary	1	2.0%
Other^†^	6	11.8%

Focusing on data specific to kidney transplants, as expected, living non-related kidney donation was the commonest source for kidney graft (94%), while 6% of the cohort received kidneys from deceased donors. Due to limited medical records provided by the patients returning after overseas transplants, we were unable to ascertain how the patients got on the waiting list of a different country, nor could we estimate the waiting time to get a deceased donor kidney transplant overseas. The pre-transplant serological testing showed that more than half of patients were positive for cytomegalovirus (CMV), and almost 90% were positive for Epstein-Barr virus (EBV). Anti-thymocyte globulin (ATG) based induction therapy was used in only 22% of patients, while the rest received a basiliximab-based regimen. Nearly all patients (98%) received a maintenance immunosuppressive regimen consisting of tacrolimus, prednisone, and mycophenolate. Regarding clinical course immediately following kidney transplant surgery, only one patient required intensive care unit admission, almost 8% developed surgical compilations (wound infection 6%, urine leak 2%), and 31% developed urinary tract infection. The value of serum creatinine at the time of discharge following kidney transplant surgery was missing in almost three-fourths of the cohort, as most of the patients receiving transplantation overseas brought limited records with them. Of those who had a creatinine reading at the time of discharge following the overseas transplant, the median value was 107 umol/l, with a maximum value of 349 umol/l recorded in one patient, and 10% of patients developed delayed graft function. No mortality occurred after one year of follow-up; however, almost 30% of patients developed graft rejection in this period. Long-term post-transplant complications were rare (diabetes mellitus 2%, malignancy 0%, psychiatric illness 2%, and fractures 4%).

When comparing the overseas transplanted cohort with their locally transplanted counterparts, we found that patients who had transplantation overseas constituted 21% of the transplant cohort followed up at KAMC during the specified period of the study. The number of patients transplanted overseas decreased by 75% between 2016 and 2018. Half of the overseas transplants occurred in 2016, followed by 11 transplants in 2017, 13 transplants in 2018, and only one in the first half of 2019. On the other hand, the number of kidney transplants performed locally increased by 48% in the same period (n=44 in 2016, n=51 in 2017, n=65 in 2018, and n=33 in 2019).

The baseline characteristics of the recipients of overseas kidney transplants were similar to our locally transplanted cohort. Although the number of patients with no comorbidities was higher in our local transplant cohort (20% vs. 10%), the difference in the number or types of comorbidities among the two groups did not reach any statistical significance (p=0.23). The type of induction therapy used did differ significantly between the two groups. The use of an ATG-based regimen was more prevalent locally compared to overseas (58% vs. 22%, p<0.001). We did not find any difference in the number or type of surgical/medical complications that developed during initial admission for kidney transplant between the two groups. In addition, the OR for delayed graft function was similar between the two groups (OR=1.5, 95% CI: 0.5-4.4, p=0.5). However, the OR for graft rejection in the first year was significantly higher in the overseas group compared to the locally transplanted cohort (OR=5.4, 95% CI: 2.4-12.0, p<0.001). When we restricted the analysis to non-related donors, the graft rejection rate remained significantly higher in the overseas transplanted group compared to the locally transplanted cohort (OR=4.1, 95% CI: 1.5-10.8, p=0.004; see Table 2 for more details).

**Table 2:** Comparison of locally transplanted and overseas transplanted cohorts MD¥ - mean difference, ATG - anti-thymocyte globulin

	Variable	Overseas transplant (n=51)	Local transplant (n=194)	MD¥	95% CI	p-value
Baseline characteristics	Mean age (years)	43.3	45	1.7	(-2.9) - 6.3	0.5
	Mean pre-transplant BMI (kg/m²)	26.5	26.1	-0.4	(-2.1) - 1.2	0.6
				p-value		
	Male	68.60%	55.70%	0.1		
	Diabetes mellitus	37.30%	34.50%	0.7		
	Hypertension	84.30%	77.30%	0.3		
	Dyslipidemia	23.50%	22.70%	0.9		
	Stroke	0.00%	2.60%	0.6		
	Cancer	2.00%	1.50%	0.8		
	Amputation	2.00%	1.50%	0.8		
	Number of comorbidities					
	No comorbidity	10.00%	20.20%	0.2		
	One	50.00%	36.30%			
	Two	18.00%	18.70%			
	Three or more	22.00%	24.90%			
Induction agent	ATG based regimen	21.60%	57.70%	<0.001		
Outcomes				OR	95% CI	p-value
	Medical complications after surgery	16 (31.4%)	53 (27.3%)	1.2	0.6-2.4	0.6
	Surgical complications after surgery	4 (7.8%)	5 (2.6%)	3.2	0.8-12.4	0.1
	Delayed graft function	5 (9.8%)	13 (6.7%)	1.5	0.5-4.4	0.5
	Graft rejection at one year	15 (29.4%)	14 (7.2%)	5.4	2.4-12.1	<0.001

The details of the graft rejection in the overseas transplanted group are presented in Table 3. 

**Table 3:** Characteristics of the overseas transplanted patients who had graft rejection AMR - antibody-mediated rejection; ATG - anti-thymocyte globulin; IVIG - intravenous immune globulin; PLEX - plasma exchange; RITUX - rituximab ^†^units in umol/l, *units in mg/kg

	Type of rejection	Class	Treatment	ATG dose*	Creatinine^†^		
					Peak	Discharge	One year
1	Cellular	IIA	ATG	4	190	139	148
2	Cellular	IIA	ATG	5	204	92	180
3	Mixed	IIB+AMR	ATG, IVIG, PLEX, RITUX	6	199	84	70
4	Cellular	IIB	ATG	5	245	90	129
5	Cellular	IA	Prednisone	n/a	346	116	114
6	Cellular	IIA	ATG	4	169	116	101
7	Cellular	IIB	ATG	5	712	157	127
8	Cellular	Borderline	Prednisone	n/a	208	145	232
9	Cellular	IIB	ATG	5	563	253	157
10	Cellular	IB	ATG	4	379	259	164
11	Cellular	IIB	ATG	4	733	243	135
12	Cellular	IIA	ATG	4	178	149	124
13	Cellular	IIA	ATG	4	196	138	101
14	Mixed	IIB+AMR	ATG, IVIG, PLEX, RITUX	7	937	180	202
15	Cellular	IB	ATG	4	149	131	118

Fifteen patients (29%) developed graft rejection; all of them were diagnosed upon the patients' first presentation to our hospital within one week of their renal transplant surgery. Thirteen patients (25%) had cellular rejection; 11 of them were treated with pulse steroid followed by a tapering steroid regimen and ATG at a dose of 4-7 mg/kg. The remaining two patients, one had borderline rejection and one had Banff IA, were treated with pulse steroids followed by a tapering dose of prednisone. Only one patient required hemodialysis for two weeks until he recovered his renal function. Two patients had mixed rejection, cellular and antibody-mediated rejection (AMR), and were treated with ATG at a dose of 6-7 mg/kg, pulse steroid followed by a tapering steroid, five sessions of plasma exchange, intravenous immunoglobulins at a dose of 100 mg/kg after each session of plasma exchange and rituximab at a dose of 375 mg/m^2^ once weekly for four doses. The mean peak creatinine at the time of rejection was 361 umol/l; upon discharge after treatment of rejection episode was 153 umol/l, and at one year was 140 umol/l. Only three patients out of those who experienced rejection had normal creatinine at one year.

## Discussion

Over the last three decades, transplant experts have expressed concerns regarding overseas transplantation practice [10,14]. In this study, we observed unfavorable outcomes in our overseas transplanted cohort, particularly the five-fold higher graft rejection rate. To attenuate the effect of HLA matching, we restricted the comparison to the recipients of unrelated kidney donation only, and the rejection rate remained higher among those transplanted overseas. Of note, none of the recipients of kidney transplants overseas was able to provide the pre-transplant HLA typing.

We also noted that the type of induction therapy varied significantly between the local and overseas groups, where patients who underwent overseas transplants were less likely to receive an ATG-based induction regimen. Our center's approach is to give ATG to the patients with high immunological risk, while patients with low immunological risk can receive basiliximab. This approach is consistent with kidney disease: Improving Global Outcomes (KDIGO) practice guidelines [15]. We hypothesized that overseas transplants might take place at centers that are not well-equipped to do high-resolution HLA typing and panel reactive antibodies (PRA), thus will inaccurately estimate the immunological risk in recipients of a kidney transplant. Moreover, overseas transplant is commercially driven, which may explain why most patients transplanted overseas tend to receive basiliximab more often as it is less costly. Giving basiliximab to patients with high immunological risk or without properly assessing the immunological risk may explain, at least in part, the higher rate of rejection among our overseas transplanted cohort [16].

The development of acute rejection of the transplanted kidney reduces long-term graft survival, particularly if not completely reversed [17,18]. It is alarming that despite our best effort to manage the acute rejection episodes, 80% did not normalize their creatinine by the time of discharge nor at one year. Moreover, patients who return to dialysis after allograft failure are at higher risk for developing inferior clinical outcomes, including mortality [19]. On this base, we recommend that transplant nephrologists counsel patients with ESRD about the risk and adverse outcomes associated with overseas transplantation, particularly the high rate of acute graft rejection.

Since overseas transplant is commercially driven, patients in overseas transplant centers tend to have the shortest possible postoperative hospital stay and are asked to fly to their home country upon discharge. The mean length of stay of our overseas transplanted group was 3.4 days. This explains why these patients present immediately to our center to establish care for their transplanted kidney and often require hospitalization for re-evaluation for any transplant-related acute complications. We found that patients who transplanted kidney overseas had higher surgical and medical complications in the immediate postoperative period compared to the locally transplanted cohort. Similarly, delayed graft function and long-term post-transplantation complications such as diabetes were also higher in this group; however, these differences did not reach statistical significance. The lack of statistical significance is likely related to the small sample size of the locally transplanted cohort. 

We hypothesize that patients who underwent transplantation may have had more comorbidities and thus were ineligible to undergo transplantation locally. However, no statistical difference was found between our locally transplanted cohort and those who had transplantation overseas in number or type of comorbidities. This supports our hypothesis that the lack of available donors was the main driver for those patients to seek kidney transplantation overseas. A recent survey showed that the availability of donors is the most common barrier to kidney transplantation [20]. Living-related donors were the main source of kidney grafts as per our local experience. Moreover, only one in five transplanted patients received their kidney from cadaveric donors [13]. Hence, it is likely that patients who are unable to find a suitable living donor may travel overseas for transplantation via a non-related living donor. We believe that national campaigns that increase awareness about organ donation after death will help increase the donor pool and thus limits the need for a patient with ESKD to travel overseas for transplant. 

A local survey revealed that only 56% of patients on dialysis were referred for pre-transplant evaluation and 29% were on the active transplant list [20]. It is possible that patients who traveled overseas for transplants were not referred to their local transplant program in a timely manner which has driven them to seek transplants overseas. Thus, we recommend that all eligible patients should be referred as soon as possible for discussion about kidney transplant. We believe that addressing these issues with adequate planning and proper actions will help curb out the practice of overseas transplantations. 

It is reassuring that the number of recipients of commercial transplants has fallen from 2016 to 2019. This may be related to the nationwide initiative that was launched in 2017 to increase public awareness of the importance of organ donation, both living and deceased organ donation. The initiative also increased the awareness of transplant tourism and its associated risks. Our previously reported local data showed that the number of kidney transplants performed at our center had gone up. However, there is still more work to be done to expand the donor pool and limit commercial transplantation. 

Our study has strengths and limitations. This study is one of the few local studies that provided a comprehensive overview of overseas kidney transplantation in Saudi Arabia. It also offered insight into the outcomes of overseas transplants compared to a reasonably matched locally transplanted cohort who underwent transplantation within the same time frame. Our findings substantiate the previously raised concerns regarding transplant tourism and identify areas of improvement to decrease the commercial transplant. The main limitation of our study is the small sample size, a single-center study, and the short follow-up period. In addition, the observational and the retrospective nature of our study made it susceptible to various types of bias. Further studies are needed to examine the long-term outcomes of transplant tourism and determine the factors that compel patients to seek commercial transplants.

## Conclusions

Commercial kidney transplantation is associated with a higher rate of rejection episodes than locally performed unrelated kidney transplantation. The use of basiliximab induction without properly assessing the pre-transplant immunological risk may explain the higher rate of rejection. Further studies are highly needed that examine the long-term outcomes of commercial transplantation and identify factors that drive such practice. Also, urgent interventions to increase the donor pool, both living and deceased, and timely referral to transplantation centers are strongly recommended to curb the need for commercial transplantation.
